# Carotid body hypoxia induces angiogenesis and correlates with glomic artery anatomy and carotid artery atherosclerosis

**DOI:** 10.1016/j.isci.2026.116482

**Published:** 2026-06-30

**Authors:** Atieh Seyedian Moghaddam, Sara Samanian Baghersad, Andreas Hainfellner, Stefan H. Geyer, Wolfgang J. Weninger

**Affiliations:** 1 Division of Anatomy, Center for Anatomy and Cell Biology, RPMI, Medical University Vienna, 1090 Vienna, Austria

**Keywords:** Cardiovascular medicine, Human Physiology

## Abstract

The carotid body (CB) monitors arterial oxygen and triggers cardiovascular and respiratory responses. We aimed to investigate how carotid artery atherosclerosis and the number and topology of glomic arteries (GAs) affect the expression of hypoxia and angiogenesis marker genes in human CBs. Thirty carotid bifurcations and CBs were harvested, and high-resolution episcopic microscopy (HREM) data volumes of the carotid arteries and GAs were created. The origin, courses, and lumen occlusion of the GAs and the grade and position of atherosclerotic plaques were evaluated. Real-time qPCR quantified hypoxia, sprouting, and intussusceptive angiogenesis marker genes expression. We demonstrate that the degree of both GA lumen occlusion and carotid artery atherosclerosis correlates with the expression levels of the hypoxia marker gene and all angiogenesis marker genes in both pathways. In addition, we define the minimal and maximal levels of hypoxia and angiogenesis marker genes in CB tissue.

## Highlights

• Carotid artery atherosclerosis drives hypoxia and angiogenesis pathways in the CB
• Glomic artery blockage and carotid plaque severity can predict hypoxia responses in the CB
• Glomic artery occlusion correlates with the upregulation of CB hypoxia and angiogenesis genes

## Graphical abstract

**Figure:**
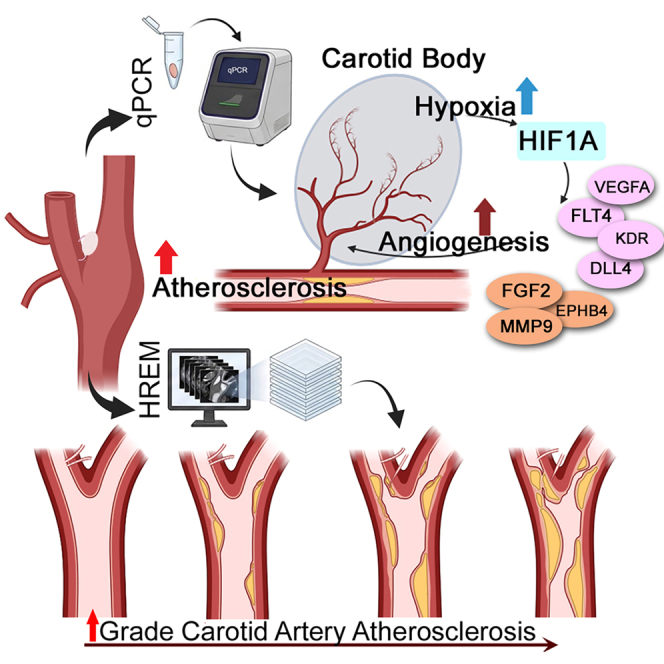

## Introduction

The carotid body (CB) is a chemoreceptor that monitors arterial oxygen levels and regulates respiration and blood pressure.^1^^,^^2^ In humans, it is located at the carotid bifurcation, and its arterial blood is supplied via one to five, in most cases two glomic arteries (GAs). They usually arise from the external carotid, but may also originate from the common carotid, carotid bifurcation, and the ascending pharyngeal artery.^3^ Reduction of blood flow in these arteries has a similar effect as decreased systemic oxygen levels and is considered to be causal to pathologies such as obstructive sleep apnea syndrome, breath alterations, hypertension, and heart failure.^1^^,^^2^

Atherosclerotic lesions arise preferentially at sites exposed to high blood pressure and disturbed blood flow,^4^ wherefore kinking, bifurcations, and blood vessel branching, especially in arteries with high blood pressure, are susceptible to atherosclerosis.^5^ Consequently, the carotid bifurcation and the adjacent segments of the carotid arteries are among the most frequently affected sites at which atherosclerotic plaques form.^6^^,^^7^^,^^8^ From biomedical model organisms, it is known that carotid artery atherosclerosis causes narrowing of GAs and diminished blood flow to the CB.^1^^,^^9^ In the rat and mouse, this results in hypoxia and enlargement of the CB.^10^^,^^11^ In humans, a relationship between carotid artery atherosclerosis and CB hypoxia has never been confirmed. Hence, one of our goals was to narrow this gap and to examine relationships between the grade and location of atherosclerotic plaques near the carotid bifurcation and CB hypoxia.

The hypoxia-induced increase in CB volume is considered to chiefly result from glomus cell proliferation.^12^^,^^13^^,^^14^ Since it is well known that hypoxia can trigger angiogenesis,^10^^,^^15^^,^^16^ we hypothesize that angiogenesis, which extends the vascular bed, might also occur in the human CB and contribute to its already described enlargement.^14^^,^^17^ One of our goals is to test whether angiogenesis is triggered in the CB by carotid artery atherosclerosis and/or GA blockade.

Angiogenesis can be triggered by the upregulation of hypoxia-inducible factor subunit alpha (*HIF-1A*), which activates one or both of the two angiogenic pathways. The first pathway, sprouting angiogenesis, causes the sprouting of new blood vessels from already-formed ones. Besides others, it involves vascular endothelial growth factor A (*VEGFA*), kinase insert domain receptor (*KDR*), fms-related receptor tyrosine kinase 4 (*FLT4*), and notch ligand delta-like 4 (*DLL4*).^18^^,^^19^ *VEGFA* binds to and activates *KDR*, which in turn triggers endothelial cell sprouting and induces *DLL4. DLL4* then activates Notch signaling in neighboring cells, which suppresses further *VEGFA* responses to control excessive branching. Finally, *FLT4* contributes to vessel stabilization and maturation.^20^

The second pathway, intussusceptive angiogenesis, causes splitting of existing vascular channels. Three prominent genes involved in this process are fibroblast growth factor 2 (*FGF2*)*,* matrix metallopeptidase 9 (*MMP9*)*, and ephrin type B receptor 4 (EPHB4).*^19^^,^^21^^,^^22^ *FGF2* stimulates the proliferation of endothelial cells, which form intraluminal tissue pillars. *MMP9* facilitates this process by degrading the extracellular matrix, and *EPHB4* guides vessel patterning and stabilizes the newly formed vessels.^20^ We decided to perform transcriptomic analysis on these genes to examine whether hypoxia triggers angiogenesis.

Traditional concepts claim that blood is supplied to the CB by a single GA, which predominantly arises from the carotid bifurcation or external carotid artery.^23^^,^^24^^,^^25^ In contrast, a recent study on 120 CBs harvested from human body donors demonstrates that regularly two and, as variations, up to five GAs supply the CB’s vascular bed. These arteries originate from the external carotid artery in approximately 60% of individuals. In 23%, they arise from the common carotid, in 10% from the ascending pharyngeal artery, and in 5% from the carotid bifurcation.^3^ If atherosclerotic plaques block a single GA, it is to be expected that the others might suffice in preventing serious hypoxia of the CB tissues. This leads to the hypothesis that hypoxia marker transcript levels in the CB correlate with the number of GAs and their potential blockade or narrowing. Furthermore, since moderate carotid artery atherosclerosis often spares the external carotid artery—from which the majority of GAs arise—it is to be expected that not all individuals with moderate atherosclerosis suffer CB hypoxia.

Despite extensive studies on the CB, the link between its microvascular architecture and genomic responses in the human CB remains largely unexplored. This is due to limited access to appropriate material and imaging technologies. Hence, the influence of carotid atherosclerosis on the blood supply to the CB and its transcriptomic response has never been examined. In the last decade, we established logistics for delivering dead body donors to our institution in postmortem time spans <10 h. In addition, we developed and optimized a high-resolution 3D imaging method, high-resolution episcopic microscopy (HREM).^26^^,^^27^^,^^28^ This permits visualizing and analyzing both the topology of the small arteries supplying the CB in the context of the large vessels they arise from^3^^,^^29^ and the extension of atherosclerotic plaque materials. We therefore decided to combine HREM imaging with transcriptome profiling of CB tissues to describe the influence of carotid arterosclerosis on GA patency and the impact of the number, topology, and occlusion of GAs on the expression of hypoxia and angiogenesis marker genes in the CB tissues.

## Results

### Atherosclerosis

Four specimens (13.33%) showed no, five specimens (16.67%) Grade 1, 11 specimens (36.67%) Grade 2, and 10 specimens (33.33%) Grade 3 atherosclerosis. Of the 26 specimens with atherosclerosis, all specimens had atherosclerotic plaques in the common and internal carotid artery, 73% had additional plaques in the external carotid artery, and 69% had additional plaques at the carotid bifurcation (Table 1).

**Table 1:** Relationship of atherosclerosis, number, and origin of glomic arteries (GAs) and hypoxia marker

CAAS Grade	Sample No	GA NO	Plaque localization				GA Orifice				HIF-1a Relative expression
			ECA	ICA	CCA	CBF	0–50% occlusion)	50–99% occlusion	100% occlusion	No occlusion	
Grade 0	1	1	–	–	–	–	–	–	–	1	0.0001
	2	2	–	–	–	–	–	–	–	2	0.0007
	3	2	–	–	–	–	–	–	–	2	0.0005
	4	2	–	–	–	–	–	–	–	2	0.0008
Grade 1	5	2	∗	∗	∗	–	–	–	–	2	0.0019
	6	2	∗	∗	∗	–	–	–	–	2	0.0022
	7	2	–	∗	∗	–	1	1	–	–	1.4597
	8	3	–	∗	∗	∗	1	–	–	2	1.3449
	9	2	–	∗	∗	–	1	–	–	1	1.3083
Grade 2	10	2	–	∗	∗	∗	1	–	–	1	1.3665
	11	1	∗	∗	∗	∗	1	–	–	–	1.4093
	12	3	∗	∗	∗	–	1	–	–	2	1.4157
	13	1	∗	∗	∗	∗	–	1	–	–	1.9796
	14	2	∗	∗	∗	–	–	1	–	1	2.1213
	15	3	–	∗	∗	∗	2	–	–	1	2.1070
	16	1	∗	∗	∗	∗	1	–	–	–	2.0726
	17	2	∗	∗	∗	∗	2	–	–	–	2.0097
	18	1	∗	∗	∗	∗	1	–	–	–	2.0105
	19	4	∗	∗	∗	∗	1	1	–	2	2.3799
	20	2	∗	∗	∗	∗	1	1	–	–	2.5863
Grade 3	21	3	∗	∗	∗	∗	–	2	–	1	5.6200
	22	2	∗	∗	∗	∗	–	1	–	1	5.6065
	23	3	∗	∗	∗	∗	–	2	–	1	5.3316
	24	2	–	∗	∗	∗	–	1	–	1	5.3167
	25	2	∗	∗	∗	–	–	–	1	1	6.0370
	26	3	–	∗	∗	–	–	1	–	2	5.3935
	27	1	∗	∗	∗	∗	–	–	1	–	8.7847
	28	2	∗	∗	∗	∗	–	–	2	–	9.0818
	29	1	∗	∗	∗	∗	–	–	1	–	9.2315
	30	1	∗	∗	∗	∗	–	–	1	–	9.5031

CAAS, carotid artery atherosclerosis; GA, glomic artery; ECA, external carotid artery; ICA, internal carotid artery; CCA, common carotid artery; CBF, carotid bifurcation.

### Number of GAs and plaque association

Eight (26.67%) CBs were supplied by one GA, 15 (50%) by two, six (20%) by three, and one (3.33%) by four GAs (Table 1). 38 GAs (63.33%) branched from the external carotid artery, 16 (26.67%) from the common carotid artery, three (5%) from the carotid bifurcation, and three (5%) from the ascending pharyngeal artery. 53% of the GAs arose from blood vessel segments affected by atherosclerosis. In 92% of the specimens with atherosclerosis, the orifice of at least one of the GAs was located inside a plaque. Table 1 provides detailed information.

### Hypoxia marker

**Figure 1:**
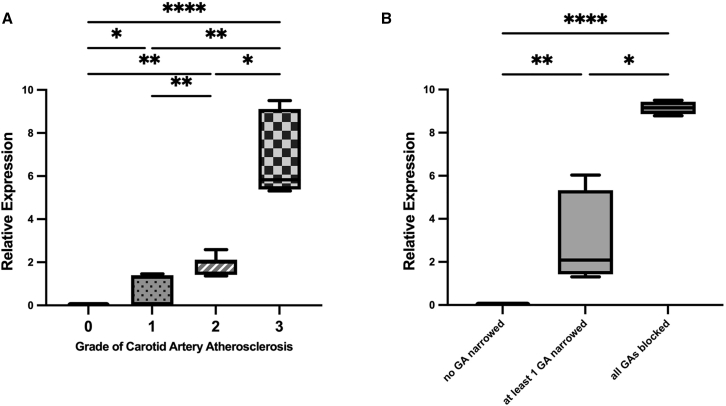
Relative expression levels of *HIF-1A* in carotid body (CB) tissue (A) Relation to the grade of carotid artery atherosclerosis (*n* ≥ 4 per group). (B) Relation to CB’s blood supply. GA, glomic artery (*n* ≥ 4 per group). Boxes show the median and interquartile range (IQR), and whiskers extend to 1.5 times the IQR. ∗*p* < 0.05, ∗∗*p* < 0.01, ∗∗∗*p* < 0.001, and ∗∗∗∗*p* < 0.0001 by Kruskal-Wallis test.

In general, the expression levels of the hypoxia marker gene *HIF-1A* in the CB tissues were closely related to the grade of carotid artery atherosclerosis (Figure 1A). All specimens without atherosclerosis and the two specimens with grade 1 atherosclerosis, which only have GAs that arose outside atherosclerotic plaques, showed *HIF-1A*-related expression below 0.003 (Table 1; Figure 1B). Specimens with grade 1 and 2 atherosclerosis having at least the orifice of one of their GAs inside an atherosclerotic plaque showed mild or severe narrowing of the lumen of this GA and relatively low (1.460–2.586), but significantly elevated *HIF-1A* expression (Table 1; Figure 1A). Specimens with grade 3 atherosclerosis exhibited high *HIF-1A* expression (5.620–9.503) (Table 1; Figure 1A). The highest *HIF-1A*-relative expression was observed in specimens with CBs that were only supplied by GAs that passed an atherosclerotic plaque and had a fully obstructed lumen (8.785–9.503) (Table 1; Figure 1B). Interestingly, as soon as at least one GA arose from a vessel segment unaffected by grade 3 atherosclerosis, the relative expression of *HIF-1A* was significantly lower (5.317–6.037) (Table 1; Figure 1B).

### Correlation between hypoxia and angiogenesis pathways

In general, the expression levels of all tested genes associated with both intussusceptive and sprouting angiogenesis show a strong positive correlation with *HIF-1A* expression (r > 0.9, *p* < 0.01) (Figure S1). Among the two angiogenesis pathways, sprouting angiogenesis-related genes showed, on average, higher expression levels than intussusceptive angiogenesis-related genes.

### Sprouting angiogenesis pathway

**Figure 2:**
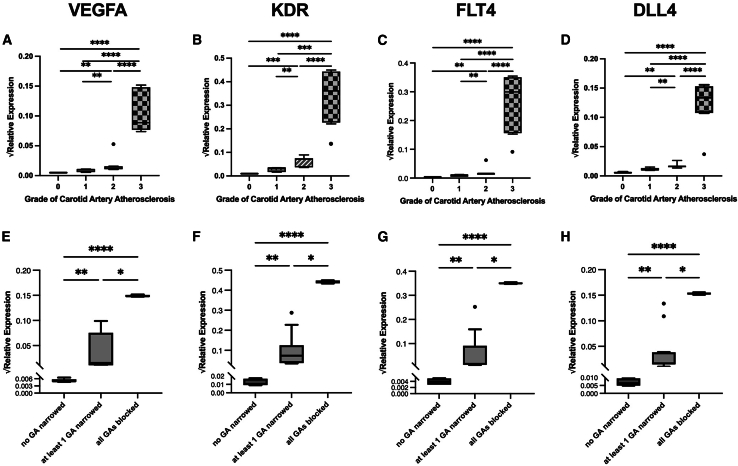
Expression levels of sprouting angiogenesis pathway genes (*VEGFA, KDR, FLT4, DLL4*) in carotid body tissue (A–D) Relation to the grade of carotid artery atherosclerosis (CAAS) (*n* ≥ 4 per group). (E–H) Relation to narrowing of the glomic arteries (GAs). (*n* ≥ 4 per group). Relative expression values are square-root-transformed (√relative expression). Boxes show the median and interquartile range (IQR), and whiskers extend to 1.5 times the IQR. ∗*p* < 0.05, ∗∗*p* < 0.01, ∗∗∗*p* < 0.001, and ∗∗∗∗*p* < 0.0001 by Kruskal-Wallis test.

The min-max relative expression (√relative expression) of all sprouting angiogenesis marker genes in specimens without atherosclerosis and in the specimens with grade 1 atherosclerosis, in which no GA was narrowed, was 0.005–0.006 for *VEGFA*, 0.009–0.017 for *KDR*, 0.003–0.005 for *FLT4*, and 0.005–0.010 for *DLL4* (Figures 2A–2H). Their expression was elevated to 0.010–0.011 in *VEGFA*, 0.034–0.035 in *KDR*, 0.011–0.013 in *FLT4*, and 0.010–0.015 in *DLL4*, in specimens with grade 1 atherosclerosis and narrowing of at least one GA (Figures 2A–2D). In specimens with grade 2 atherosclerosis, they were elevated to 0.010–0.053 in *VEGFA*, 0.036–0.089 in *KDR*, 0.012–0.062 in *FLT4*, and 0.014–0.026 in *DLL4*. In those with grade 3 atherosclerosis, they were elevated to 0.074–0.152 in *VEGFA*, 0.119–0.450 in *KDR*, 0.088–0.354 in *FLT4*, and 0.037–0.156 in *DLL4* (Figures 2A–2D). In this group, the specimens in which grade 3 atherosclerosis fully blocked all GAs, the values were by far the highest, with 0.147–0.152 in *VEGFA*, 0.434–0.450 in *KDR*, 0.347–0.354 in *FLT4*, and 0.151–0.156 in *DLL4* (Figures 2E–2H).

**Figure 3:**
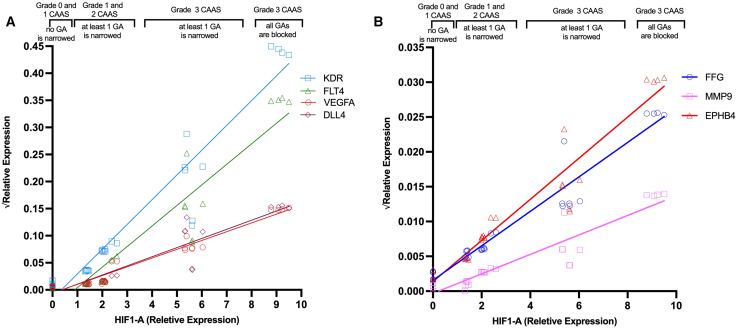
Correlation of the expression level of hypoxia (*HIF1-A*) and angiogenesis marker genes (A) Sprouting angiogenesis (*VEGF, KDR, FLT4, DLL4*) marker genes (*n* ≥ 4 per group). (B) Intussusceptive angiogenesis (*FFG, MMP9, EPHB4*) marker genes (*n* ≥ 4 per group). GA, glomic artery; CAAS, carotid artery atherosclerosis. *q* < 0.001, by FDR-adjusted Spearman’s correlation coefficient (r).

Correlation analysis showed a strong correlation of all the sprouting angiogenesis marker genes with *HIF-1A* expression levels (Figure 3A).

### Intussusceptive angiogenesis pathway

**Figure 4:**
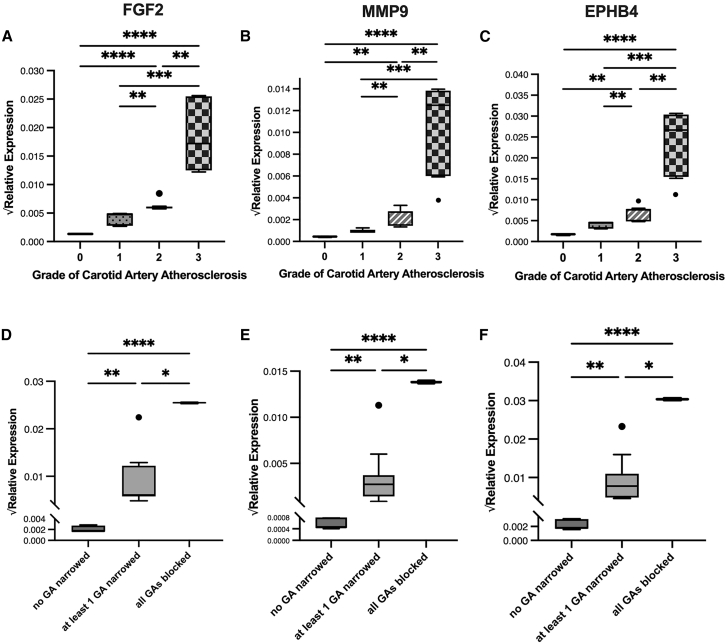
Expression level of intussusceptive angiogenesis pathway genes in carotid body tissue (A–C) Relative expression of intussusceptive angiogenesis pathway genes (*FGF2, MMP9, and EPHB4*) in different grades of carotid artery atherosclerosis (CAAS) (*n* ≥ 4 per group). (D–F) Relative expression of all the intussusceptive angiogenesis genes in relation to the association of the GAs with atherosclerotic plaque (*n* ≥ 4 per group). Relative expression values are square-root-transformed (√relative expression). Boxes show the median and interquartile range (IQR), and whiskers extend to 1.5 times the IQR. ∗*p* < 0.05, ∗∗*p* < 0.01, ∗∗∗*p* < 0.001, and ∗∗∗∗*p* < 0.0001, by Kruskal-Wallis test.

The min-max relative expression (√relative expression) of all intussusceptive angiogenesis marker genes in specimens without atherosclerosis and in specimens in which no GA was narrowed was 0.002–0.003 in *FGF2*, 0.0004–0.0008 in *MMP9*, and 0.002–0.003 in *EPHB4* (Figures 4A–4F). Their expression was elevated to 0.0048–0.0049 in *FGF2*, 0.0009–0.0013 in *MMP9*, and 0.0045–0.0047 in *EPHB4*, in specimens with grade 1 atherosclerosis and narrowing of at least one GA (Figures 4A–4C). In specimens with grade 2 atherosclerosis, they were elevated to 0.006–0.008 in *FGF2*, 0.001–0.003 in *MMP9*, and 0.005–0.011 in *EPHB4* (Figures 4A–4C). In those with grade 3 atherosclerosis, they were elevated to 0.012–0.026 in *FGF2*, 0.004–0.014 in *MMP9*, and 0.011–0.031 in *EPHB4* (Figures 4A–4C). In this group, the specimens in which grade 3 atherosclerosis fully blocked all GAs, the values were by far the highest, with 0.025–0.026 in *FGF2*, 0.137–0.140 in *MMP9*, and 0.030–0.031 in *EPHB4* (Figures 4D–4F).

Correlation analysis showed a strong correlation of all intussusceptive angiogenesis marker genes with *HIF-1A* expression levels (Figure 3B).

## Discussion

In this study, we combined an advanced high-resolution digital 3D imaging technique, HREM, with transcriptomic profiling of human CB tissues. The great morphological detail of the digital datasets and the use of body donor material harvested at <10h postmortem allowed linking the expression of hypoxia- and angiogenesis-related genes in the CB with its arterial supply and with carotid artery atherosclerosis. The materials examined were derived from both sides of 15 body donors who had died between 71 and 97 years (Table S1). Since we had no records providing information on carotid atherosclerosis in these specimens, we also used HREM for the diagnosis and for the criteria suggested by the American Society of Echocardiography (ASE)^29^ for grading carotid artery atherosclerosis. In 16.67% of carotid arteries, we observed grade 1 atherosclerosis, in 36.67% grade 2, and in 33.33% grade 3. These frequencies were consistent with findings already reported for older adults.^30^

The large-field HREM data volumes had a resolution of 3.8 × 3.8 × 3.0 μm^3^. This permitted the creation of high-detailed volume-rendered models of the atherosclerotic plaques in the blood vessels of the carotid bifurcations, and at the same time, the generation of surface-rendered models of the very small GAs. A total of up to four GAs were identified. 63% arose from the external carotid artery and the rest from the common carotid, the carotid bifurcation, and the ascending pharyngeal arteries. These results are in line with a recently published large-scale study that systematically investigated the number and origins of the GAs in an elderly central European population.^3^

All specimens were harvested within 10 h after death (Table S1). It is to be expected that in such a short period, the CB tissues have not yet fully adapted their transcriptomes to postmortem conditions. Indeed, we were able to isolate sufficient amounts of RNA from all CBs to allow analyses of the level of *HIF-1A* transcripts with real-time qPCR. Since we analyzed specimens with fully blocked GAs and specimens only supplied via fully patent GAs, we were able to define the minimal and maximal levels of each transcript.

In 12 out of the 15 body donors, the number and occlusion of the GAs and/or the grade of atherosclerosis differed between the right and left side (Table S1). This prevented us from comparing individuals and forced us to consider each CB as an independent experimental unit.

Due to the peculiarities of the human vascular system, the heart pumps blood almost directly to the carotid bifurcation, which causes high levels of wall pressure and heterogeneous shear stress at this site.^31^ Disturbed shear stress induces endothelial dysfunction characterized by increased endothelin-1 expression and activation of endothelin receptor signaling,^32^^,^^33^ which promotes inflammatory and oxidative stress pathways and contributes to atherosclerotic plaque development.^34^ The progression of plaques leads to the expression of *HIF-1A*. *HIF-1A* then triggers *VEGF*-mediated angiogenic responses.^35^^,^^36^ Since the carotid bifurcation and the proximal internal carotid artery are prone to showing atherosclerotic alterations,^37^ we hypothesized that plaques, located near the orifices of the GAs, challenge CB perfusion and cause hypoxia-driven angiogenesis in the CB. The low *HIF-1A* transcript levels in the specimens with normal CB blood supply, and the very high levels in the specimens with fully blocked GAs, support this assumption. Hence, we consider the measured levels of the hypoxia marker gene as closely representing the situation before death. The measurements obtained from the six specimens, in which all GAs arose from sites with normal blood vessel walls, therefore represent “baseline” or background under study conditions (Table 1; Figure 1A), and √ relative expression of 0.005–0.006 for *VEGFA*, 0.009–0.017 for *KDR*, 0.003–0.005 for *FLT4*, 0.005–0.010 for *DLL4,* 0.002–0.003 for *FGF2*, 0.0004–0.0008 for *MMP9*, and 0.002–0.003 for *EPHB4* (Figures 2E–2H; Figures 4D–4F). On the other hand, relative expression levels of 8.785–9.503 for *HIF-1A* (Table 1, Figure 1B) and the √ relative expression levels of 0.147–0.152 for *VEGFA*, 0.434–0.450 for *KDR*, 0.349–0.354 for *FLT4*, 0.151–0.156 for *DLL4*, 0.025–0.026 for *FGF2*, 0.013–0.014 for *MMP9*, and 0.030–0.031 for *EPHB4* (Figures 2E–2H; Figures 4D–4F), as measured in the specimens, in which all GAs were fully blocked, can be considered as the maximum expression levels of these genes in human CB tissue under study conditions.

Together, these findings may be interpreted as indicating that the CB retains a transcriptional profile reflective of pre-mortem hypoxic adaptation. Notably, even when only one out of several GAs was mildly narrowed, we observed an increase in *HIF-1A* transcripts. Since the expression levels of angiogenesis markers strongly correspond to the hypoxia levels, we speculate that the vascular bed of the CB is highly dynamic and the CB constantly responds to changing conditions by regulating the extension of its vascular bed according to its needs.

Furthermore, the highest levels of *HIF-1A* transcripts were observed in the four specimens, in which all GAs passed through an atherosclerotic plaque and were fully blocked. These specimens were derived from two body donors. Unfortunately, we did not have access to the full medical records of these individuals. However, the high transcript levels of angiogenesis markers suggest that even these individuals had vivid CB tissues, or at least only moderate RNA fragmentation, despite the intense hypoxia. We speculate that necrosis was prevented by the blood supply to the CB by local anastomoses at the capillary level.

Our data show that the elevation of *HIF-1A* transcripts in the CB induces both the elevation of sprouting and intussusceptive angiogenesis marker gene transcripts (Figures 3A and 3B). Interestingly, only patients with grades 2 and 3 carotid atherosclerosis develop hypertension.^38^ We speculate that the angiogenic response to minor hypoxia, as caused by grade 1 atherosclerosis, is sufficient to secure the maximum supply of CB cells with oxygen and thus normal CB function. Upon entering grade 2, this fails, and even increased blood supply by angiogenesis is no longer able to provide sufficient amounts of oxygen to the glomus cells.

In conclusion, we defined minimal and maximal transcript levels of hypoxia and angiogenesis marker genes in the human CB and demonstrated that the grade of carotid artery atherosclerosis is associated with GA number and occlusion, and elevated expression of hypoxia, sprouting, and intussusceptive angiogenesis marker transcripts. This elevation correlates with the grade of atherosclerosis and with GA narrowing by plaque material. High angiogenesis marker transcript levels in the CB tissue of individuals with fully blocked GAs may suggest adaptive vascular remodeling; however, functional validation at the protein and microvascular levels is required to verify or falsify this. Since we had to use all CB tissues for transcriptome analysis, no tissue was available for such additional examinations. Future studies are therefore required to research adaptive vascular remodeling, but also to research the real effects of the CB changes on circulatory and respiratory function.

### Limitations of the study

Sample size: Restrictions in the availability of tissue material and financial considerations forced us to examine only 30 specimens. As a consequence, the total number of 30 specimens is high enough for significant statements considering the total cohort. However, numbers in subgroups are relatively low. Nevertheless, even these findings draw a clear picture of the correlations between the degree of atherosclerosis and GA occlusion, and the hypoxia and angiogenesis marker transcripts in the CB.

Availability of material: Human CBs have a very small volume. We were therefore forced to restrict the analysis of the CB tissues to transcriptome analysis. Additional immunohistochemistry or other protein-level analyses were not possible.

Use of body donors: Firstly, body donors available for research usually die a natural death. Hence, the tissues we harvested are derived from persons of advanced age. Secondly, many body donors die in hospice or at home. We were therefore unable to obtain their full medical records. Thirdly, almost all body donors enrolled in our body donor program lived in Central Europe. In a strict sense, our findings therefore only describe the situation in a Central European population.

## Resource availability

### Lead contact

Requests for further information and resources should be directed to and will be fulfilled by the lead contact, Atieh S. Moghaddam (atieh.seyedianmoghaddam@meduniwien.ac.at).

### Materials availability

This study did not generate new, unique reagents.

### Data and code availability

• Data reported in this paper will be shared by the lead contact upon request.
• This paper does not report original code.
• Any additional information about the data reported in this paper is available from the lead contact upon request.

## Acknowledgments

The authors sincerely thank the individuals who donated their bodies to science, making this research possible. We extend our deepest gratitude to these donors and their families. Special thanks are also extended to Sylvia Gerges and Laurin Schatzer for their technical assistance.

## Author contributions

A.S.M.: conceptualization and study design, specimen collection, data acquisition, data analysis, data interpretation, writing the manuscript, and preparing the figures. S.S.B.: conceptualization, genomic data acquisition, and writing the manuscript. A.H.: specimen collection. S.H.G.: data acquisition and analysis and critical revision. W.J.W.: conceptualization, study design, supervision, project administration, supervision of manuscript writing, and assembling. All authors reviewed the manuscript.

## Declaration of interests

The authors declare that no third-party funding was received for the experiments, and none of the authors has a financial interest in any of the devices and products mentioned in this manuscript.

## STAR★Methods

### Key resources table

**Table:** 

REAGENT or RESOURCE	SOURCE	IDENTIFIER
**Chemicals, peptides, and recombinant proteins**		

QIAzol Lysis Reagent	Qiagen	79306

**Oligonucleotides**		

*GAPDH* RT-qPCR primers	Microsynth	See Table S3
*VEGFA* RT-qPCR primers	Microsynth	See Table S3
*KDR* RT-qPCR primers	Microsynth	See Table S3
*FLT4* RT-qPCR primers	Microsynth	See Table S3
*DLL4* RT-qPCR primers	Microsynth	See Table S3
*FGF2* RT-qPCR primers	Microsynth	See Table S3
*MMP9* RT-qPCR primers	Microsynth	See Table S3
*EPHB4* RT-qPCR primers	Microsynth	See Table S3
*HIF-1A* RT-qPCR primers	Microsynth	See Table S3

**Software and algorithms**		

AMIRA	Thermo Fisher Scientific, Waltham, MA, USA	Version 2020, RRID:SCR_007353

GraphPad Prism	GraphPad Software, Inc. Sandiego, CA, USA	Version 10.0.3 (217), RRID:SCR_002798
R	R Foundation for Statistical Computing, Vienna, Austria	R Core Team (2024), RRID:SCR_001905
BioRender	https://BioRender.com	©2026 BioRender

### Experimental model and study participant details

The study was carried out at the Division of Anatomy, Medical University of Vienna. Human body donors enrolled in the local body donor program were used in accordance with the Declaration of Helsinki. All body donors had voluntarily signed informed consent that their dead bodies are used for education and science. In addition, the study was approved by the Ethics Committee of the Medical University of Vienna (Ethics number 1359/2024).

A total of 15 body donors were included in this study (Table S1). The age at death ranged from 71 to 97 years (mean: 83.5 years), comprising 10 males and 5 females. All donors had a Central European background. Although both sexes were included, the study was not powered to detect sex-specific differences.

### Method details

#### Sample collection

30 carotid bifurcations with CBs and the associated distal-most segments of the common carotid and the proximal-most segments of the internal and external carotid arteries were harvested from 15 individuals (five females, ten males). All samples were harvested within the first 10 h after death according to established protocols.^39^ None of the body donors had scars in the thorax and neck or a confirmed history of CB tumor. Demographic and non-demographic data are summarized in Table S1.

In all bodies, the carotid triangle was bilaterally exposed by employing traditional anatomic approaches and using anatomic instruments.^40^ The nerves supplying the CB and the common, external, and internal carotid arteries were cut at a distance of 2 cm from the carotid bifurcation. The specimens were extracted and placed in cold PBS. The CBs were separated from the vessels, snap-frozen in liquid nitrogen, and stored in a −80°C freezer until being processed for transcriptome analysis. The remaining artery segments were transferred into 4% paraformaldehyde (PFA) for fixation. Dissection and specimen preparation were done while wearing gloves and using sterile instruments.

#### Tissue preparation for HREM

All fixed artery samples were kept in PFA until they were prepared for digital volume data generation with the high-resolution episcopic microscopy (HREM) technique according to modified standard protocols.^41^^,^^42^

In brief, the PFA fixed samples were rinsed in running tap water for 3 h and dehydrated in increasing ethanols (50%, 5 h; 70%, overnight; 80%, 90%, and 96%, 5 h each) at 4°C. Following dehydration, they were infiltrated for 14 days in JB-4 solution A of a resin embedding kit (Polysciences, Warrington, PA, USA), to which 0·4g eosin and 1 · 25g catalyst (benzoyl peroxide, plasticized) per 100 mL had been added. The solution was changed three times. The infiltrated specimens were placed in embedding molds and embedded in a freshly prepared infiltration solution, to which 1 mL JB-4 solution B (accelerator) per 25 mL JB-4 solution A had been added. After placing block holders on the embedding molds, they were sealed airproof, and the blocks were allowed to harden for two days. Finally, the blocks were extracted and baked at 70°C–80 °C for a minimum of 1–2 days to finish polymerization.^43^

The hardened resin blocks were mounted on an HREM apparatus and sectioned in 2–3 μm steps. The resulting series of 6000–6500 digital images per sample were transferred to a PC workstation equipped with 192 GB RAM and an NVIDIA Titan XP graphics card. They were virtually stacked to form digital volume data using the Amira software 2020® (Thermo Fisher Scientific, Waltham, MA, USA) and handled as described earlier.^44^ Voxel dimensions were 3.8 × 3.8 × 3.0 μm^3^.

#### HREM data analysis

Using the data processing and analysis tools of Amira®, the HREM data were displayed and analyzed using volume rendering and virtual dissection algorithms. The number and origins of the GAs were recorded, and their orifices were checked for luminal narrowing according to established criteria.^45^ They were classified on the percent of lumen occlusion: no occlusion, 0–50% occlusion, 50–99% occlusion, and 100% occlusion. The occlusion was measured on cross-sectional planes through the orifices of the GAs. The carotid bifurcations were screened for atherosclerotic plaques. If present, their precise positions and relations to the GAs were recorded. Finally, the plaques were graded following the criteria suggested by the American Society of Echocardiography (ASE) (Grade 0: No plaque; Grade 1: plaque thickness <1.5 mm; Grade 2: plaque thickness between 1.5 and 2.4 mm; Grade 3: plaque thickness ≥2.5 mm) (Figure 5).^46^

**Figure:**
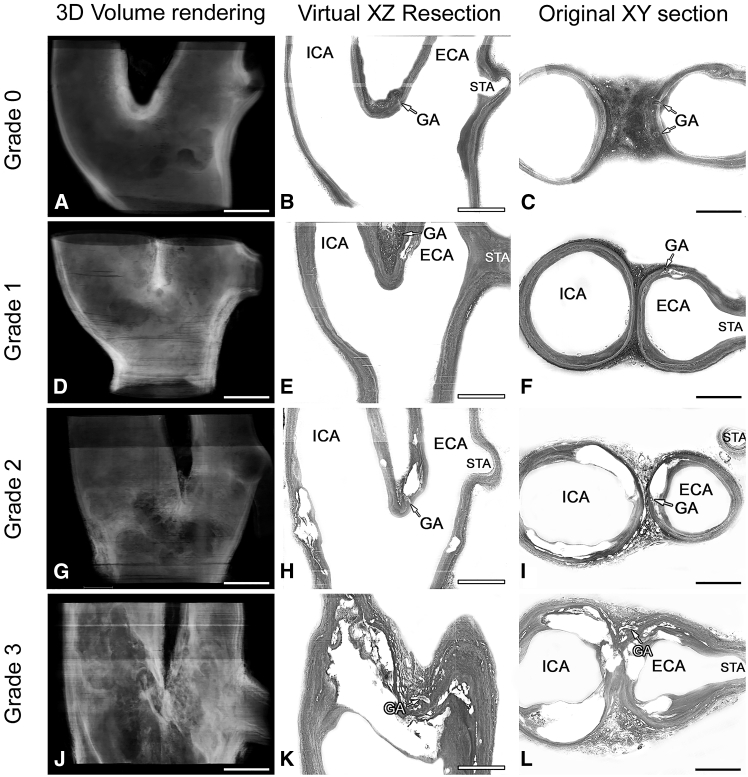
Grading of carotid artery atherosclerosis digital volume data generated with high-resolution episcopic microscopy (HREM) The carotid body is removed. (A–C) No plaque. (D–F) Grade 1. (G-I) Grade 2. (J–L) Grade 3. Semitransparent HREM volume renderings of the carotid bifurcation and associated vessel segments from anterior (A, D, G, J). Virtual coronal resection through HREM volume data (B, E, H, K). Original axial HREM sections at the level of carotid bifurcation (C, F, I, L). Note the relation of plaque tissue and glomic arteries (GAs). ECA: external carotid artery; ICA: internal carotid artery; STA: superior thyroid artery. Scale bars, 3000 μm.

#### Gene expression analysis

Snap-frozen tissue samples were homogenized under sterile conditions using FastPrep-24^TM^. RNA was extracted employing QIAzol Lysis Reagent (Qiagen, 79306). In order to remove possible DNA contamination, DNase treatment was conducted using the Qiagen RNase-Free DNase Set (Qiagen, 79254) according to the manufacturer’s instructions. The concentration and purity of RNA samples were measured using a NanoDrop- 2000 (Thermo Fisher, MA, US).

1000 ng of extracted RNA was reverse transcribed into cDNA using M-MuLV reverse transcriptase (New England Biolabs GmbH, M0253L), and random hexamers (NEB, S1330S). RT-qPCR amplifications were performed utilizing Luna Universal qPCR Master Mix (New England Biolabs GmbH, M3003×) with the CFX384 Touch Instrument (Bio-Rad, US).

The specific primers for *GAPDH, VEGFA, KDR, FLT4, DLL4, FGF2, MMP9, EPHB4,* and *HIF-1A* were designed using Primer3 based on the respective mRNA sequences retrieved from NCBI and considered to be exon-exon junctions (Table S3). Cycling conditions for RT-qPCR were: 95°C for 1 min (1×); 95°C for 15 s followed by 60°C for 30 s (40×). All samples were run in duplicate.

The 2^−ΔCt^ method was employed to calculate the relative expression level of genes in target samples, with normalization performed using GAPDH.

### Quantification and statistical analysis

Statistical analysis was performed using GraphPad Prism (Version 10.0.3 (217); GraphPad Software, Inc. Sandiego, CA, USA) and R software (R Core Team (2024). R: A language and environment for statistical computing. R Foundation for Statistical Computing, Vienna, Austria). The normality of all continuous variables was checked with the Shapiro-Wilk test. The Kruskal-Wallis test was used to compare gene expression levels among the carotid artery atherosclerosis classes and to compare the expression level of genes among CBs supplied by GAs of various levels of obstruction. Pairwise comparisons were performed using the Mann-Whitney U test with Bonferroni correction. Correlation analysis between variables was performed using Spearman’s rank correlation test (r). *p*-values were adjusted using the Benjamini–Hochberg false discovery rate (FDR) method implemented in R. The relative expression values for sprouting and intussusceptive angiogenesis genes were square-root transformed to minimize heteroscedasticity.

In 12 out of 15 body donors, the grade of carotid artery atherosclerosis, the number of GAs supplying the CB, and the degree of occlusion of the GA orifices differed between the left and right side (Table S2). Hence, for statistics, each CB was considered as a distinct and independent experimental unit. Statistical analyses were performed without donor-level clustering.
