# Supplementary material for: Carotid body hypoxia induces angiogenesis and correlates with glomic artery anatomy and carotid artery atherosclerosis
Source: iScience. 2026 Jun 30;29(7):116482. doi: 10.1016/j.isci.2026.116482 (PMC13343058; doi:10.1016/j.isci.2026.116482)
Supplement: Document S1. Figure S1 and Tables S1–S3 [file mmc1.pdf]

iScience, Volume *29*

Supplemental information

Carotid body hypoxia induces angiogenesis and correlates with glomic artery anatomy and carotid artery atherosclerosis

Atieh Seyedian Moghaddam, Sara Samanian Baghersad, Andreas Hainfellner, Stefan H. Geyer, and Wolfgang J. Weninger

Supplementary Table 1. Characteristics of processed samples

Variables	Subgroups	Number of body donors	%
**Sex**	Male	10	66.7
	Female	5	33.3
**Harvest time**	6-7h post mortem	4	26.7
	7-8h post mortem	6	40.0
	8-9h post mortem	3	20.0
	9-10h post mortem	2	13.3
**Age**	70-79 years	3	20.0
	80-89 years	10	66.7
	90-99 years	2	13.3

Supplementary Table 2. Intraindividual left/right differences in number and occlusion of glomic arteries (GA) and carotid artery atherosclerosis (CAAS)

Body Donor	Grade of CAAS		Number of GAs		GA occlusion							
					no occlusion		0-50%		50-99%		100%	
	L	R	L	R	L	R	L	R	L	R	L	R
1	0	0	1	2	1	2						
2	0	0	2	2	2	2						
3	1	1	2	2	2	2						
4	1	1	2	3		2	1	1	1			
5	1	2	2	2	1	1		1	1			
6	2	2	2	1	1		1	1				
7	2	2	3	1	2		1			1		
8	2	2	3	1	1		2	1				
9	2	2	2	1			2	1				
10	2	2	4	2	2		1	1	1	1		
11	3	3	3	2	1	1			2	1		
12	3	3	3	2	1	1			2	1		
13	3	3	2	3	1	2				1	1	
14	3	3	1	2							1	2
15	3	3	1	1							1	1

Note that 12 body donors (highlighted in blue) have right–left differences in number and/or occlusion of GAs and/or degree of atherosclerosis. L, left; R, right.

Supplementary Table 3. Primer sets used for real-time qPCR assays

Gene	Forward primer (5′-3′)	Reverse primer (5′-3′)	PCR- product size (bp)	Related Pathway
GAPDH	CAAGAGCACAAGAGGAAGAGAG	CTACATGGCAACTGTGAGGAG	102	-
VEGFA	GGAGGGCAGAATCATCACGA	GGCACACAGGATGGCTTGAA	140	SA
KDR	CCAGCAAAAGCAGGGAGTCT	GGAGTACACGGTGGTGTCTG	101	SA
FLT4	GGAGACAAGGACAGCGAGGA	TAGTAGCAGACGTAGCTGCC	128	SA
DLL4	ACAACCTGATTCCTGCCG	CTCCTAAGCTCTTGTCACTGTG	189	SA
FGF2	TGTGTGCTAACCGTTACCTGG	TCGTTTCAGTGCCACATACCA	158	IA
MMP9	CAGAGATGCGTGGAGAGTCG	AGGTTTGGAATCTGCCCAGG	156	IA
EPHB4	CTGGCTTCGCACAGGTT	TCCGCATCGCTCTCATAGTA	150	IA
**HIF-1A**	TGAAACGCCAAAGCCACTTC	CCTGAATCTGGGGCATGGTA	119	Hypoxia

SA, sprouting angiogenesis; IA, intussusceptive angiogenesis.

Supplementary Fig. 1. Correlation heat map of gene expression levels among genes

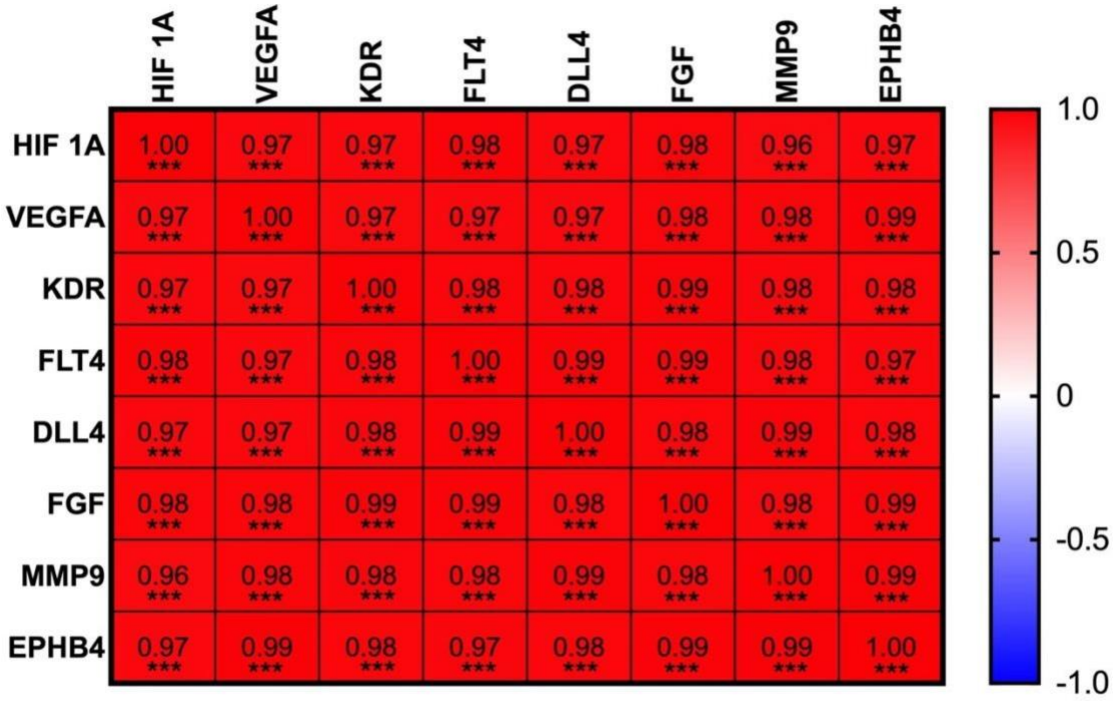

FDR-adjusted Spearman's correlation coefficient (r). All correlations are significant at the 0·01 level (2-tailed). FDR adjusted q value after Benjamin-Hochberg correction: * q <0.05, ** q < 0.01, *** q < 0.001. (n=30 per group).
